# GMI ablates cancer stemness and cisplatin resistance in oral carcinomas stem cells through IL-6/Stat3 signaling inhibition

**DOI:** 10.18632/oncotarget.19711

**Published:** 2017-07-31

**Authors:** Tung Yuan Wang, Cheng-Chia Yu, Pei-Ling Hsieh, Yi-Wen Liao, Chuan-Hang Yu, Ming-Yung Chou

**Affiliations:** ^1^ School of Dentistry, Chung Shan Medical University, Taichung, Taiwan; ^2^ Department of Dentistry, Chung Shan Medical University Hospital, Taichung, Taiwan; ^3^ Institute of Oral Sciences, Chung Shan Medical University, Taichung, Taiwan

**Keywords:** oral squamous cell carcinomas, immunomodulatory protein GMI, cancer stem cells, chemoresistance, IL-6/Stat3 signaling

## Abstract

Cancer stem cells (CSCs) have been identified to exert tumor-initiating ability, resulting in the recurrence, metastasis and chemoresistance of oral squamous cell carcinomas. In the present study, we showed that GMI, an immunomodulatory protein from *Ganoderma microsporum*, induc ed a cytotoxic effect in oral carcinomas stem cells (OCSCs). Treatment of GMI dose-dependently inhibited the expression of CSC markers, including ALDH1 activity and CD44 positivity. Moreover, GMI suppressed the self-renewal property, colony formation, migration, and invasion abilities as well as potentiated chemo-sensitivity in OCSCs. Our results suggested that the tumor suppressive effect of GMI was mediated through inhibition of IL-6/Stat3 signaling pathway. Furthermore, tumor growth was reduced in mice bearing xenograft tumors after oral administration of GMI. Taken together, we demonstrated the anti-CSC effect of GMI in oral cancer and GMI may serve as a natural cisplatin adjuvant to prevent cancer recurrence.

## INTRODUCTION

Head and neck squamous cell carcinoma (HNSCC) is one of the most common cancers worldwide and the majority of HNSCC is oral squamous cell carcinoma (OSCC) [1]. Although there has been substantial progress in cancer treatment, survival rates have improved only frugally over the past few decades [2]. A significant number of patients still suffer from recurrent cancer after chemotherapy [3]. It has been shown that 3-year overall survival rate of advanced OSCC patients with recurrence was less than 30% [4]. Accumulating evidence has suggested that the resistance of radiochemotherapy is attributed to the cancer stem cells (CSCs) within the heterogeneous tumor mass [5]. It has been indicated that CSCs exhibit stemness and are highly tumorigenic, leading to the relapse, metastasis and therapeutic refractoriness of tumor [6]. Recently, several reports have revealed that treatments against CSCs improve the efficacy of chemotherapy [7]. As such, strategy that targets CSCs may be a promising approach for OSCC.

Lingzhi, *Ganoderma lucidum*, is a medicinal mushroom used in traditional medicine as an herbal remedy for centuries. The therapeutic properties have been reported for Lingzhi species, such as anti-inflammation [8], anti-cancer [9] and immunomodulation [10]. It contains a variety of bioactive molecules for these effects, including triterpenoids, polysaccharides and fungal immunomodulatory proteins (FIPs) [11]. To date, various FIPs have been identified to show anti-tumor, anti-metastatic and anti-chemoresistant capacities, such as Lingzhi-8 from *Ganoderma lucidum* [12, 13] or FIP-gts from *Ganoderma tsuga* [14, 15]. Currently, another FIP from *Ganoderma microsporum* called GMI has been shown to possess anti-cancer effect via induction of autophagy in lung cancer cells [16, 17] and have the potential against chemo-resistance [18]. Nonetheless, the role of GMI as an anti-tumor agent to treat other type of cancer has not been examined and the signal pathways involved in this process are not well understood yet.

In this study, we assessed the effect of GMI on the stemness features and cisplatin resistance in oral cancer stem cells (OCSCs). GMI significantly suppressed the expression of CSC markers, self-renewal, clonogenic, migration and invasion abilities as well as tumorigenecity *in vivo*. And administration of GMI enhanced the tumor sensitization to chemotherapy. Moreover, we showed that tumor inhibitory effect of GMI was via IL-6/Stat3 signaling pathway. Altogether, we demonstrated that therapeutic effect of GMI in oral cancer was through the inhibition of CSCs and GMI may act as a potential cisplatin adjuvant for OSCC.

## RESULTS

### The cytotoxicity effect of GMI in oral cancer stem cells (OCSCs)

**Figure 1:**
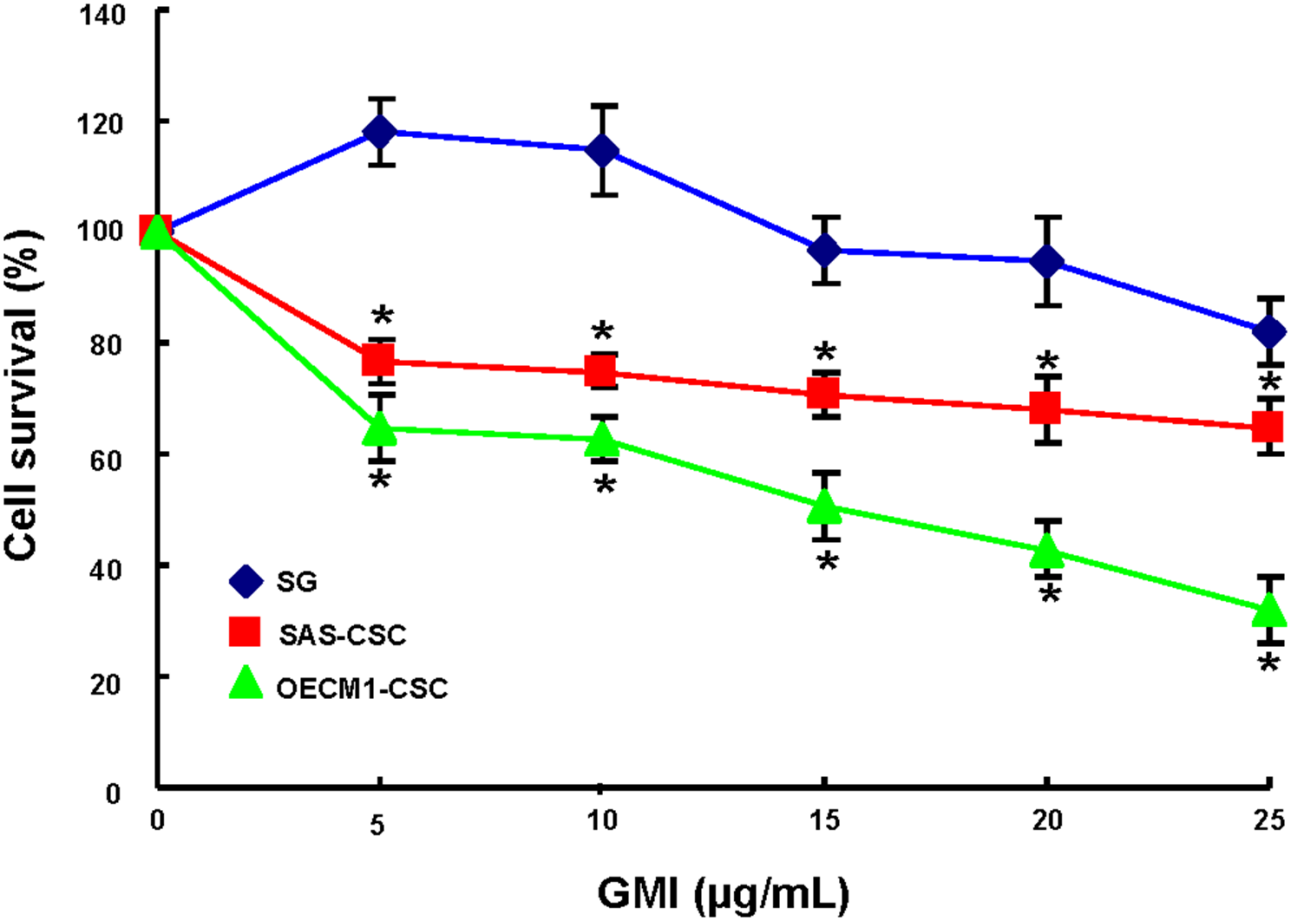
The cell viability effect of GMI in normal epithelial cells and OCSC SG and OCSC were plated in wells of 96-well-plate as 1×10^4^ cells/well in 0.1 % DMSO or different concentration of recomGMI-containing medium and cultured at 37°C for 24hr. Cell proliferation/survival was determined by MTT (3-(4,5-dimethylthiazol-2-yl)-2,5-diphenyl tetrazolium bromide) assay. The 570 nm absorbance of DMSO treated group was set as 100% and data were presented as percentage of DMSO control. Results are means ± SD. *, p<0.05 vs. Control.

We first investigated the cell survival of these two OCSCs derived from oral cancer cell lines and normal human gingival epithelioid cell line (SG) in order to understand the cytotoxicity of GMI. We treated these cells with increasing concentrations of GMI for 24 hours followed by MTT assay. As shown in Figure 1, GMI markedly suppressed the cell viability of two OCSCs without causing toxic damage to normal cells.

### GMI attenuates ALDH1 activity and CD44 positivity in OCSCs

**Figure 2:**
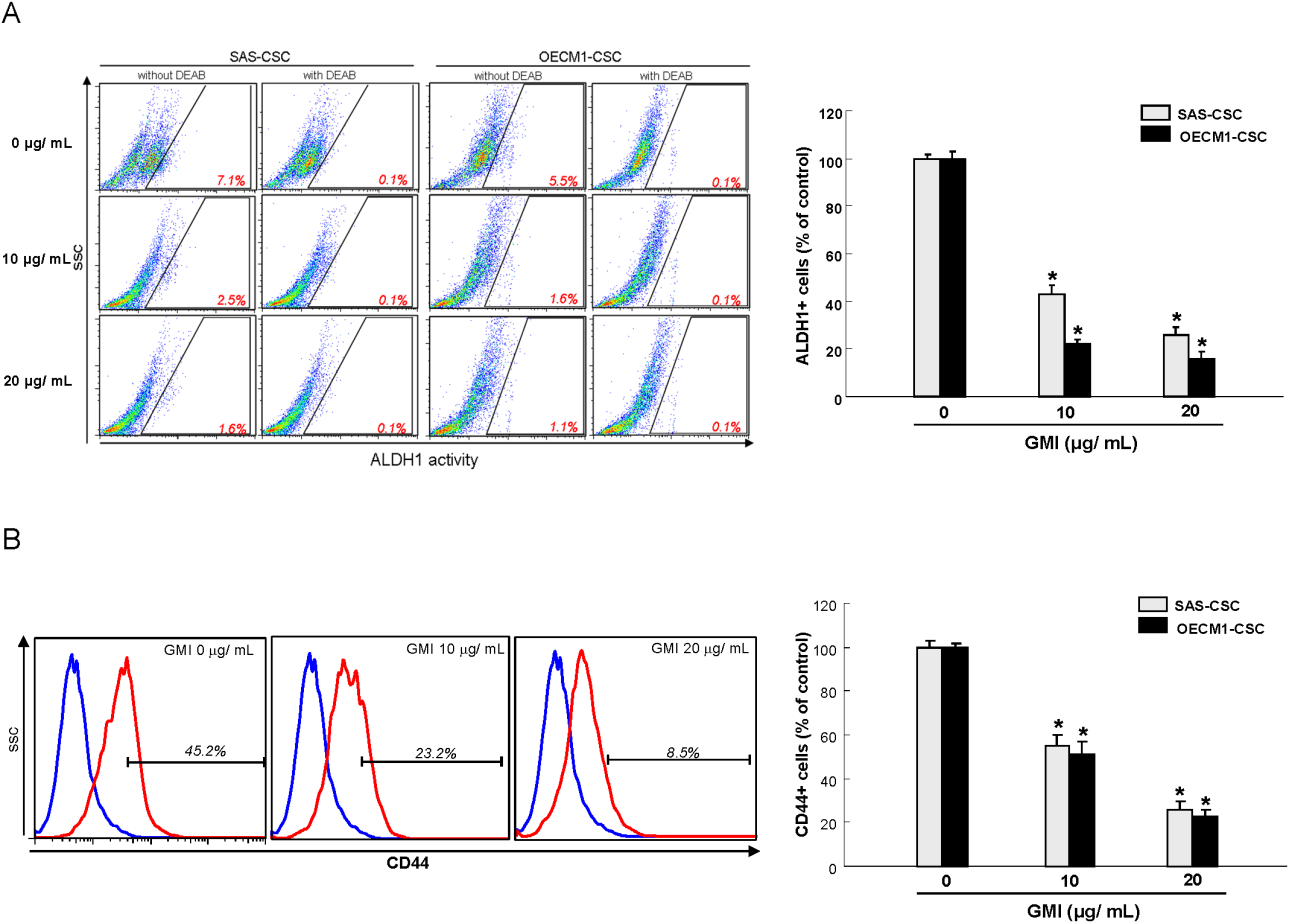
GMI effectively eliminates ALDH1 activity and CD44 positivity **(A)** For this assay, 1×10^5^ cells were suspended in 50 ml of assay buffer, and ALDEFLUOR was added to the cell suspensions for a final concentration of 1 mM. For ALDH1 inhibitor control, DEAB was added to a final concentration of 150 mM. Cells were then incubated at 37°C for 45 min and were stained with 7-AAD on ice for 5 min. After washing the cells with PBS, live cells (7AAD-) positive for green fluorescence were analyzed by flow cytometry (FACSCalibur™, BD Biosciences) to compare the fluorescence intensity of the DEAB-treated samples. High fluorescence was associated with high ALDH activity (ALDH+ cells). **(B)** Cells were stained with anti-CD44 antibody conjugated to phycoerythrin (Miltenyi Biotech., Auburn, CA, USA), with labeling according to the manufacturer’s instructions. Red (>650 nm) fluorescence emission from 10,000 cells illuminated with blue (488 nm) excitation light was measured with a FACSCalibur (Becton Dickinson) using CellQuest software. Results are means ± SD. *, p<0.05 vs. Control.

ALDH1 enzymatic activity [19] and CD44 positivity [20] have been proven to be highly selective markers for CSCs in OSCC. Our data suggested administration of GMI significantly resulted in a concentration-dependent decrease in ALDH1 activity of both OCSCs (Figure 2A). Likewise, the expression of CD44 was gradually downregulated in both OCSCs along with the increase in GMI concentration using flow cytometry analysis (Figure 2B). These results showed that GMI eliminated the proportion of OCSCs in a dose-dependent manner.

### GMI represses self-renewal and oncogenicity abilities in OCSCs

**Figure 3:**
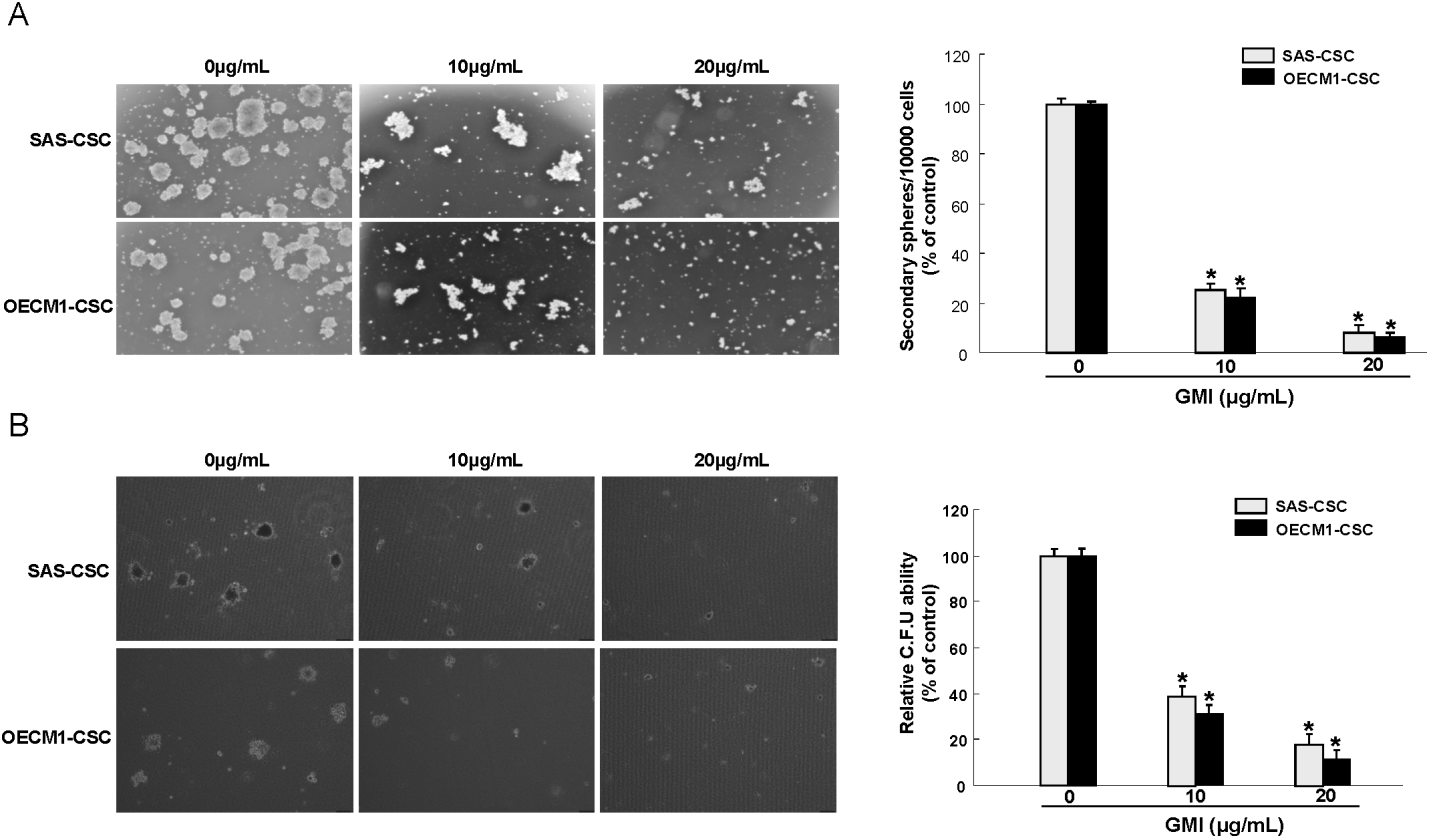
Inhibition of self-renewal property and clonogenicity in OCSC under GMI treatment **(A)** Secondary sphere formation ability of GMI-treated cells was examined. The bar graph shows quantification of secondary sphere number. **(B)** OCSC with dose-dependent GMI treatment were assigned for the colony formation assay. The experiments were repeated three times and representative results were shown. Results are means ± SD. *, p<0.05.

**Figure 4:**
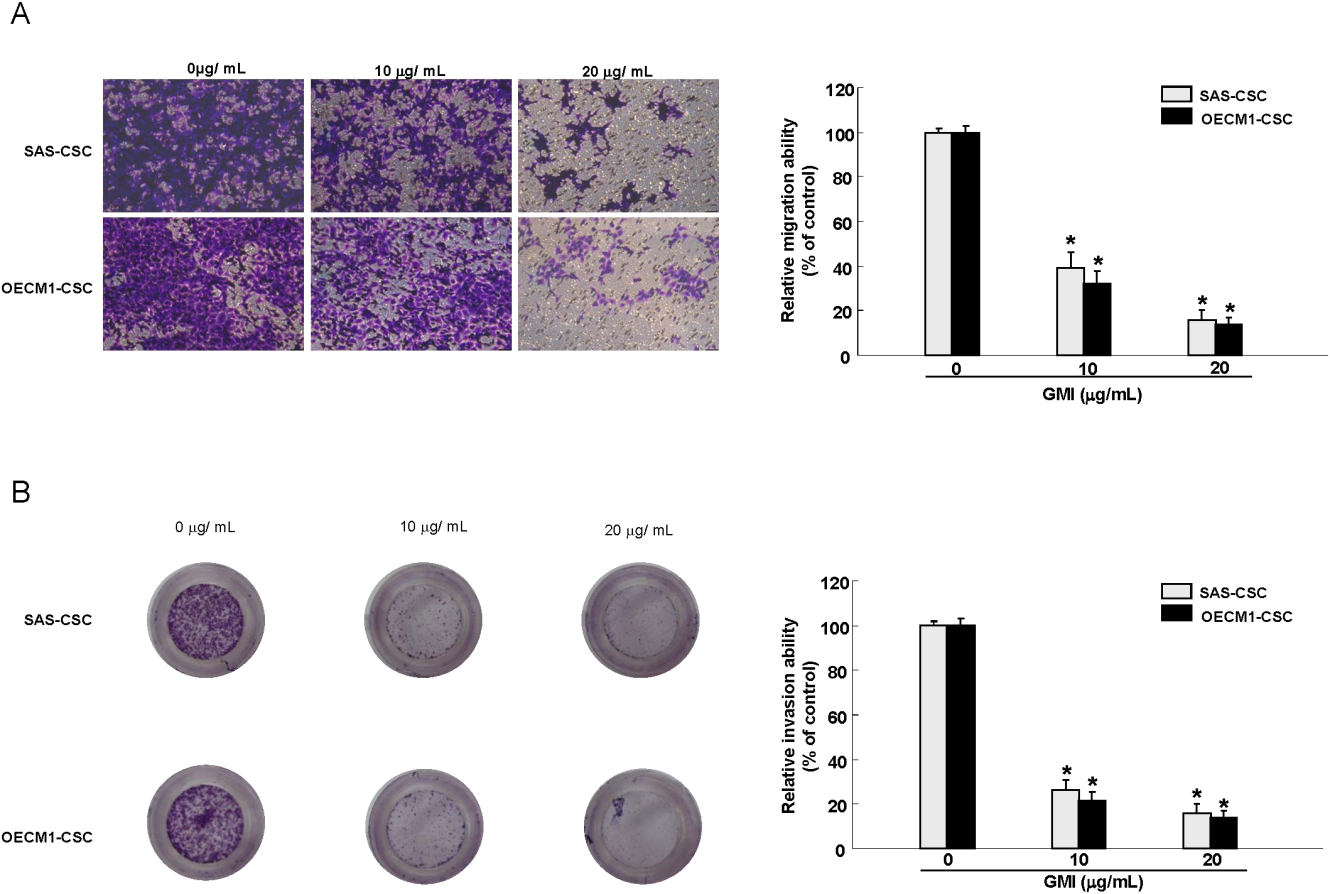
GMI abrogates migration and invasion capacity of OCSC Representative images (left) and quantification (right) of **(A)** migration assay and **(B)** Matrigel invasion assay of OCSCs treated with various concentration of GMI. Experiments were performed in triplicate. Values are expressed as mean ±SD. * *p* < .05 compared to control.

Given that CSCs exhibit highly tumor-initiating ability and are involved in metastasis and therapeutic refractoriness of cancer [6], we sought to examine the efficacy of GMI on self-renewal, colony forming, migration and invasion abilities of OCSCs. Secondary sphere formation and anchorage-independent growth assays were used to evaluate the self-renewal and colony formation capacities, respectively. A dose-dependent suppression of tumorigenic activities, including self-renewal (Figure 3A), colony formation (Figure 3B), migration (Figure 4A), invasion (Figure 4B) properties of OCSCs was observed. Collectively, these results clearly demonstrated that GMI exerted a pronounced anti-tumor effect on OCSCs.

### Enhanced chemosensitivity in OCSCs by GMI

**Figure 5:**
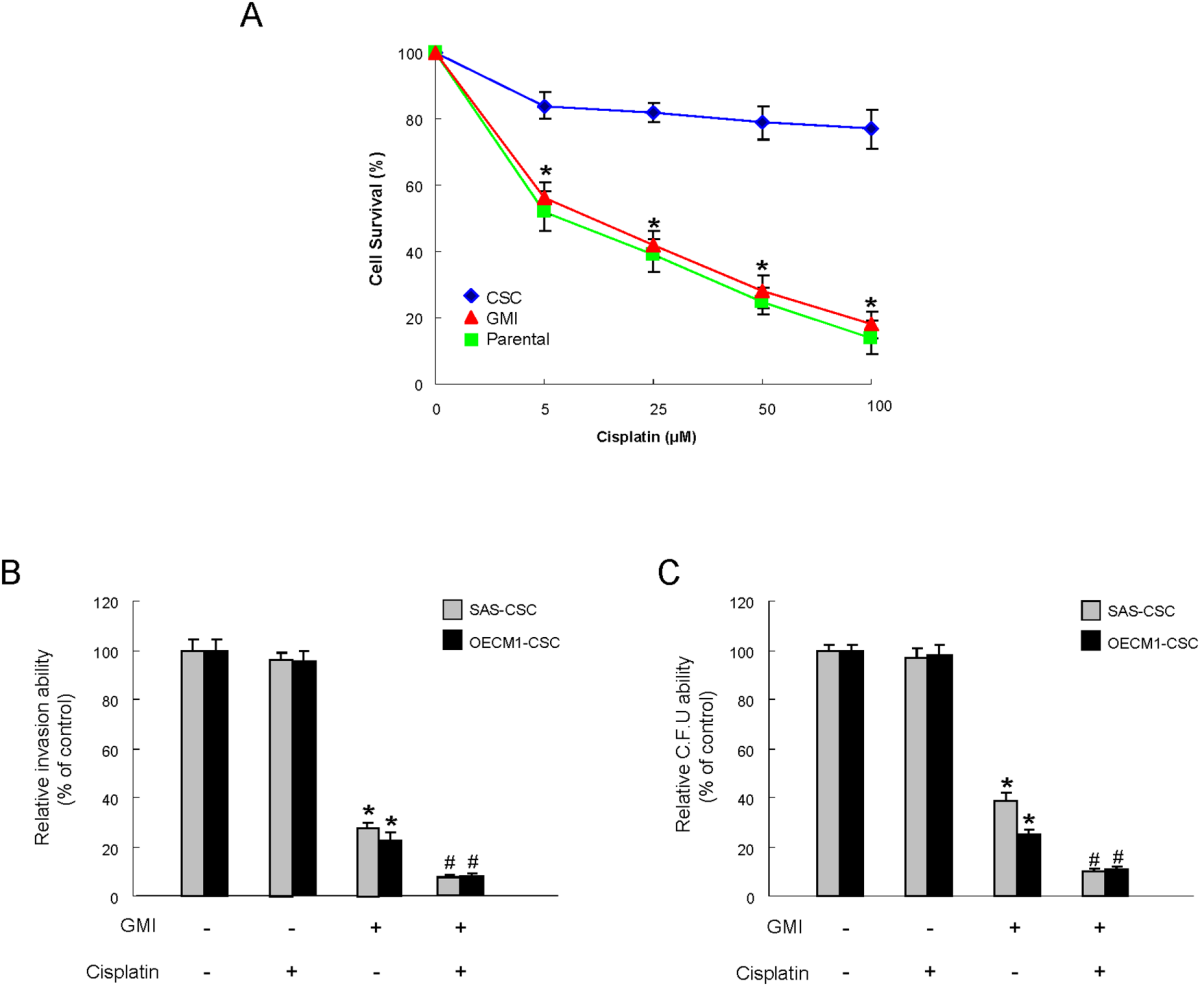
Enhanced sensitivity of OCSC by GMI treatment **(A)** After exposure to different doses of cisplatin, the surviving cell fractions of the control or GMI-treated OCSC were evaluated. **(B)** Invasion ability and **(C)** colony-forming ability in OCSC were examined after treatment with either GMI or cisplatin treatment or both. *, p<0.05 GMI vs. control; #, p<0.05 GMI+cisplatin vs. GMI alone.

The recurrence of cancers has been attributed to chemo-resistant CSC after conventional treatments [21], hence it is crucial to assess the chemosensitivity when evaluating the anti-tumor effect of GMI. As expected, drug-resistance was obviously more prevalent in OCSCs compared with parental OSCC cells using cell viability assay. Nevertheless, the sensitivity to Cisplatin in OCSC was dramatically improved in combination with GMI (Figure 5A). Moreover, the invasive (Figure 5B) and colony formation (Figure 5C) capacities were further abolished by Cisplatin in conjunction with GMI compared to GMI or Cisplatin alone. Overall, these findings demonstrated the enhanced anti-OCSC activity via the synergic action of GMI and Cisplatin.

### The anti-tumor effect of GMI is via inhibition of IL6/ Stat3 axis

**Figure 6:**
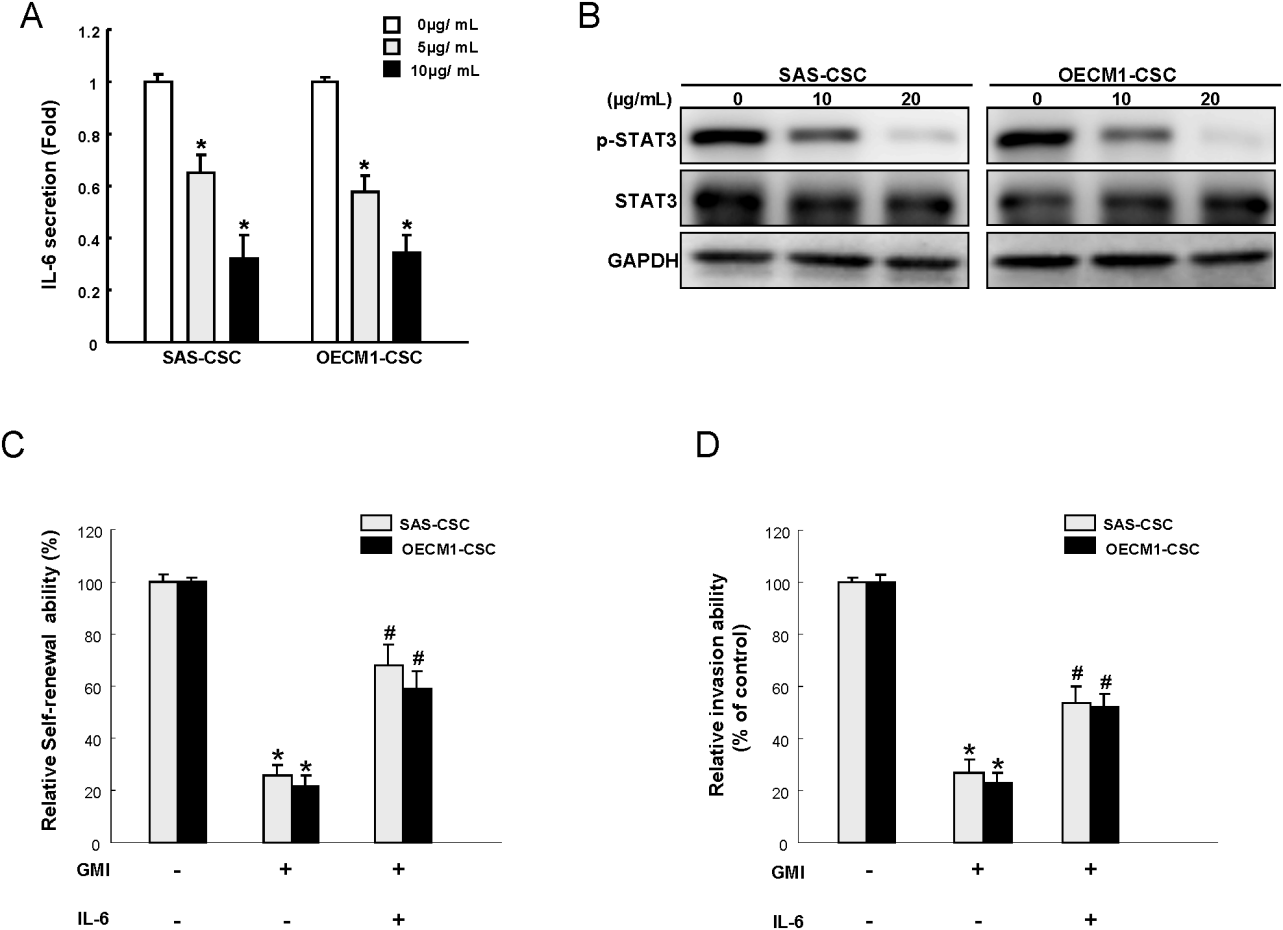
GMI treatment represses IL-6/STAT3 signaling of OCSC **(A)** IL-6 secretion level in GMI-treated OCSC was analyzed by ELISA analysis. **(B)** Cell extract proteins of GMI-treated OCSC were collected and analyzed by immunoblotting against anti-p-STAT3, anti-STAT3, or anti-GAPDH antibodies as indicated. The immunoactive signal of GAPDH protein of different crude cell extracts was referred as loading control. Self-renewal **(C)** and invasion ability **(D)** in OCSC were analyzed after treatment with either GMI treatment or GMI combined IL-6 treatment. *, p<0.05 GMI vs control; #, p<0.05 GMI+IL-6 vs. GMI alone.

The elevated expression of IL-6 has been found to be involved in erlotinib resistance of OSCC [22] and the immunomodulatory effect of GMI has been shown in a previous report [23]. In the current study, the expression of IL-6 in OCSCs was reduced by GMI in a dose-dependent fashion (Figure 6A). Since IL-6 is a major autocrine/paracrine factor to activate Stat3 in OSCC [24], we examined the expression level of Stat3 following treatment of GMI to verify the possible mechanism. Results from western blotting showed that the protein expression of phosphorylated-Stat3 was gradually down-regulated in the presence of increased GMI concentration, whereas the level of total Stat3 was not affected (Figure 6B). Furthermore, the inhibitory effect of GMI on self-renewal (Figure 6C) and invasive (Figure 6D) properties was reverted by addition of IL-6, indicating the anti-OCSCs potential of GMI was via modulation of IL-6/Stat3 axis.

### Administration of GMI exerts a suppressive effect on tumor growth *in vivo*

**Figure 7:**
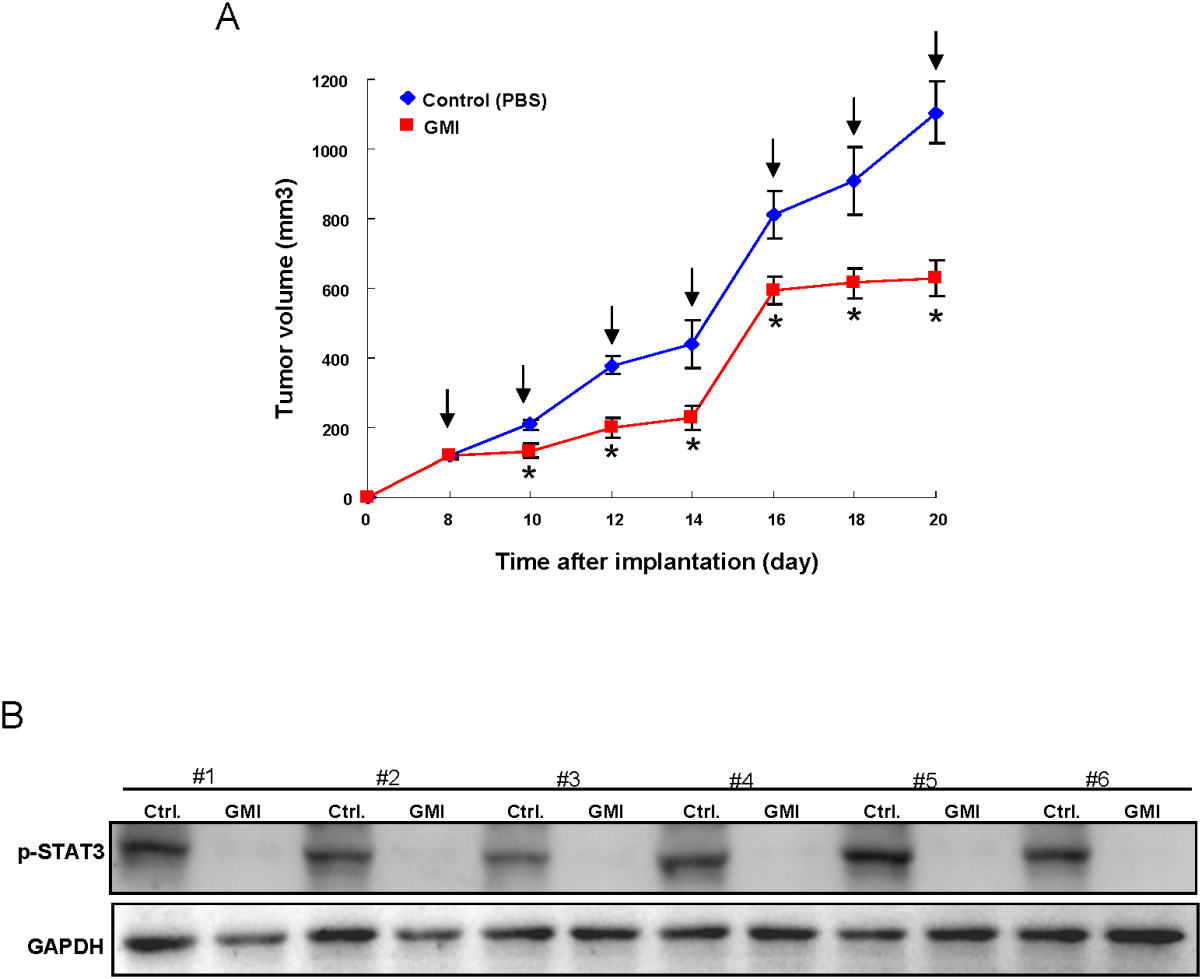
Therapeutic delivery of GMI in OCSC-transplanted mice attenuates tumor progression *in vivo* **(A)** After subcutaneous implantation of OCSC, BALB/c nude mice (*N* = 6 for each group) were oral-feeding treated with GMI and then photographed and analyzed for the and tumor volume. **(B)** Mice were sacrificed, and tumor sections as indicated treatments were assessed for p-Stat3 expression by western bloting analysis. Values are expressed as mean ±SD. * *p* < .05 compared to control.

To validate the anti-tumorigenic efficacy of GMI *in vivo*, immunocompromised mice bearing OCSC xenografts received GMI treatment or vehicle by oral gavage followed by analyses of tumor volume and expression of phosphorylated-Stat3 in tumors. As shown in Figure 7A, the tumor growth was significantly delayed after treatment of GMI. And the GMI-induced downregulation of phosphorylated-Stat3 (Figure 7B) was confirmed by western blotting.

## DISCUSSION

Over the past few decades, increasing attention has been paid to the use of natural products against cancer and it has been suggested that immunological approaches targeted CSCs may be a new direction of cancer therapy [25]. Previously, the effects of immunomodulatory protein GMI have been examined in various studies. It has been shown that GMI suppressed the EGF-mediated migration and invasion via blockage of PI3K/Akt pathway [17] and the TNF-α-induced tumor invasion and inflammation through inhibition of NF-κB/MMP-9 pathway [23] in human alveolar epithelial A549 cells. Later, the anti-tumor effect of GMI in non-small cell lung cancer cells was further investigated and proved to be mediated through activation of autophagy [16]. In addition, GMI was showed to enhance Cisplatin-induced apoptosis through the inhibition of Akt/mTOR pathway [18, 26]. These results suggested that the effects of GMI are associated with the regulation of PI3K/Akt/mTOR signaling pathway. In fact, this pathway has been shown to play a crucial role in CSC therapy [27] and it interacts with NFκB, which is integrated into essential aspects for oncogenesis [28, 29] and contributed to CSC phenotype [30].

In the current report, we revealed the novel anti-CSCs properties of GMI and demonstrated that it potentiated the effectiveness Cisplatin-based therapy for OSCC treatment. Our findings suggested that the tumor suppressive effect of GMI was via inhibition of IL-6/Stat3 signaling. Various studies have shown that hyperactivation of Stat3 is implicated in tumor progression and treatment resistance [31, 32] and Stat3 blockade enhances the efficacy of chemotherapeutic agents in OSCC [33, 34]. Stats can be activated by a variety of signal transduction pathways, such as epidermal growth factor receptor (EGFR), nicotinic receptor and interleukin (IL) receptor pathways [32]. And it has been demonstrated that IL-6 is the major autocrine/paracrine factor for Stat3 activation in OSCC [24]. Numerous studies have shown that targeting of IL-6/STAT3 signaling inhibited CSCs *in vitro* and *in vivo* [35, 36]. Consistent with these studies, we demonstrated that administration of GMI could serve as a therapeutic approach to inhibit IL-6/Stat3 signaling, thereby attenuating cancer stemness, invasiveness and chemo-resistance.

In conclusion, these data demonstrated the anti-OCSC effect of GMI *in vitro* and *in vivo.* And the tumor suppressive effect of GMI was via regulation of IL-6/Stat3 pathway. Furthermore, GMI was a chemosensitizing agent and could use as a Cisplatin adjuvant to reduce cancer recurrence. This work provided new insight into the utilization of GMI as an anti-cancer agent for OSCC therapy.

## MATERIALS AND METHODS

### Reagent and cell culture

GMI, manufactured by Mycomagic Biotechnology Co., Ltd. (Taipei, Taiwan), was generated and ameliorated from *Ganoderma microsporum.*The CSCs derived from OSCC cell lines SAS and OECM-1 as well as normal human gingival epithelioid cell line (SG) were cultivated as previously described [37].

### MTT assay

Cell viability was determined using MTT (Sigma, St. Louis, MO) to evaluate the cytotoxicity of GMI. Cells were seeded in 24-well plates (1 × 10^4^ cells/ well) in the presence of various concentration of GMI or vehicle at 37°C for 24 hours followed by incubation with MTT reagent. The blue formazan crystals of viable cells were dissolved in DMSO and then evaluated spectrophotometrically at 570 nm. DMSO-treated group was set as 100%, and data were presented as percentage of DMSO control.

### Flow cytometry analysis

For cell surface marker analysis, cells were stained with anti-CD44 antibody conjugated with phycoerythrin (Miltenyi Biotech., Auburn, CA, USA). And ALDEFLUOR kit (Stem Cell Technologies, Durham, NC, USA) was used to examine the ALDH1 enzymatic activity according to manufacturer's instructions. Fluorescence emission from 10,000 cells was measured with FACSCalibur (Becton Dickinson, Mountain View, CA, USA) using CellQuest software.

### Secondary sphere formation assay

Cells were dissociated and cultured in the modified DMEM/F-12 supplemented with N2 (R&D Minneapolis, MN, USA), 10 ng/mL epidermal growth factor (Invitrogen, Carlsbad, CA, USA), 10 ng/mL basic fibroblast growth factor (Invitrogen, Carlsbad, CA, USA), and penicillin/streptomycin at 10^3^ live cells/low-attachment six-well plate (Corning Inc., Corning, NY, USA). Medium was changed every other day until the secondary sphere formation was observed in about 2 weeks. Cell density/ 10,000 cells were presented as the percentage of control.

### Soft agarose assay

Each well of a six-well culture dish was coated with 1 ml of bottom agar (Sigma-Aldrich) mixture (DMEM/F-12, 15% (v/v) FBS, 0.525% (w/v) agar). After the bottom layer was solidified, 1 ml of top agar-medium mixture (DMEM/F-12, 15% (v/v) FBS, 0.3% (w/v) agar) containing 4 × 10^4^ cells was added, and the dishes were incubated at 37°C for 2 weeks. Plates were stained with 0.01% Crystal Violet, and then the colonies were counted.

### Cell invasion and migration assays

The 24-well plate Transwell system with a polycarbonate filter membrane of 8-μm pore size (Corning, United Kingdom) was employed to evaluate the migration and invasion abilities of cells. The membrane was coated with Matrigel (BD Pharmingen, NJ, USA) for invasion. The cell suspensions were seeded to the upper chamber of the Transwell insert within serum-free medium at the cell density of 5 × 10^4^ and 1 × 10^5^ for migration and invasion assays, respectively. The lower chamber was filled with media supplemented with 10% serum. After 24 hours of incubation, the filter membrane was stained with crystal violet (Sigma-Aldrich). The migrated and invasion cancer cells were then visualized and counted from five different visual areas of 100-fold magnification under an inverted microscope.

### ELISA analysis

For detection of IL-6, cells were cultured in 6-well plates with various concentration of GMI for 24 hours. Cell supernatants were collected and centrifuged to remove dead cells. The supernatants were then analyzed by ELISA using IL-6 specific kit from eBioscience according to the manufacturer’s instructions.

### Western blot analysis

Cell protein extraction and immunoblotting analysis were performed as previously described [38]. Briefly, sample was boiled at 95°C for 5 minutes and separated on 10% SDS-PAGE. The proteins were transferred to Hybond-ECL nitrocellulose paper or Polyvinylidene difluoride membrane (Amersham, Arlington Heights, IL, USA). The primary antibodies used were rabbit anti–Stat3, anti-phospho-Stat3 (cell signaling Santa Cruz Biotechnology) and mouse anti–GAPDH (Chemicon, Temecula, CA, USA). Immunoreactive protein bands were detected by the ECL detection system (Amersham Biosciences Co., Piscataway, NJ, USA).

### Measurement of tumor growth *in vivo*

All procedures involving animals were conducted in accordance with the institutional animal welfare guidelines of the Chung Shan Medical University. 5–6 weeks old immuno-deficient nude mice (BALB/c nu/nu mice) were used for the xenograft model. OCSC (1 × 10^4^ cells/0.2 mL/mouse) were injected subcutaneously into the right axilla and the day of cell implantation was designated as day 0. Mice were randomly divided into two groups and fed with either saline (control) or GMI (150 μg/day/kg) by oral gavage 8 days post implantation. Tumor size measurement was performed using an IVIS50 animal imaging system (Xenogen Corp.). The volume was calculated (according to the following formula: [length Χ width^2^]/2), and then analyzed by Image-Pro Plus software. After 20 days, animals were euthanized followed by tissue excision for phosphor-Stat3 analysis.

### Statistical analysis

SPSS software (version 13.0; SPSS, Inc., Chicago, IL, USA) was used for statistical analysis. The presented results are representative of three independent experiments with similar results. Statistical differences were evaluated with the Student t test, and were considered significant at *p* < 0.05.
